# Early- versus late-onset gastroesophageal cancer: real-world outcomes from a 13-year central European cohort study

**DOI:** 10.1007/s10120-026-01724-z

**Published:** 2026-03-27

**Authors:** Tomas Sokop, Iveta Selingerova, Vaclav Jedlicka, Lumir Kunovsky, Igor Kiss, Radka Lordick Obermannova

**Affiliations:** 1 Department of Comprehensive Cancer Care, Masaryk Memorial Cancer Institute, Zluty Kopec 7, Brno, 656 53 Czech Republic; 2 Department of Comprehensive Cancer Care, Faculty of Medicine, Masaryk University, Brno, Czech Republic; 3 Department of Clinical Trials, Masaryk Memorial Cancer Institute, Brno, Czech Republic; 4 Department of Mathematics and Statistics, Faculty of Science, Masaryk University, Brno, Czech Republic; 5 Department of Pharmacology and CREATIC, Faculty of Medicine, Masaryk University, Brno, Czech Republic; 6 Department of Surgical Oncology, Masaryk Memorial Cancer Institute, Brno, Czech Republic; 7 Department of Surgical Oncology, Faculty of Medicine, Masaryk University, Brno, Czech Republic; 8 Department of Gastroenterology and Digestive Endoscopy, Masaryk Memorial Cancer Institute, Brno, Czech Republic; 9 Department of Internal Medicine – Gastroenterology and Geriatrics, Faculty of Medicine and Dentistry, University Hospital Olomouc, Palacky University Olomouc, Olomouc, Czech Republic; 10 Department of Surgery, Faculty of Medicine, University Hospital Brno, Masaryk University, Brno, Czech Republic

**Keywords:** Gastric cancer, Esophageal cancer, Europe, Adult, Treatment outcome

## Abstract

**Background:**

Gastroesophageal cancer presents variably across different age groups, with early-onset (EO) cases showing distinct pathological and clinical characteristics compared to late-onset (LO) disease. This study aims to delineate these differences and assess treatment outcomes in a Central European population.

**Methods:**

Data from patients diagnosed with gastroesophageal carcinoma and treated between 2010 and 2022, at a high-volume comprehensive cancer center representing approximately 5% of all national gastroesophageal cancer cases in the Czech Republic, were retrospectively analyzed. Patients were categorized into EO (< 50 years) and LO (≥ 50 years) groups. Clinicopathological characteristics were compared in the entire cohort, while treatment patterns and survival outcomes were evaluated in patients with adenocarcinoma.

**Results:**

A total of 1,377 patients were included, with 161 (11.7%) classified as EO. EO patients are more frequently present with ECOG performance status 0 (42%), lower BMI, current smoking (44%), gastric cancer primaries (63%), and aggressive tumor characteristics such as poorly cohesive adenocarcinoma subtype (45%) and metastatic disease (55%). Hereditary cancer syndrome was confirmed in 4.3% of EO cases. Among adenocarcinoma patients, triplet chemotherapy was more frequently used in the EO group (29% vs. 11%, *p* < 0.001) in the first-line setting. However, no significant survival benefit was observed in inoperable or metastatic disease (median overall survival 9.1 vs. 9.6 months in EO and LO, respectively, *p* = 0.913).

**Conclusions:**

EO gastroesophageal cancer in the Central European population is associated with distinct clinicopathological characteristics, with no significant impact on survival.

**Miniabstract:**

This study identifies distinct clinicopathological characteristics in early-onset gastroesophageal cancer in Central Europe, characterized by more aggressive attributes but without significant survival differences compared to late-onset cases.

**Supplementary Information:**

The online version contains supplementary material available at 10.1007/s10120-026-01724-z.

## Introduction

Gastric cancer ranks fifth among solid tumors affecting adolescents and young adults (AYAs), with a global burden estimated at 1,570,000 disability-adjusted life years (DALYs) [1]. Recent data from the SEER program indicate a rising incidence of early-onset (EO) gastric cancer, defined using a 60-year age threshold, along with an increased incidence of esophageal adenocarcinoma, as a less common subtype [2]. Contrastingly, the Czech Republic’s more socio-demographically homogeneous population exhibits a different trend; data from the National Cancer Registry indicate a consistent decline in both incidence and mortality rates for EO gastric and esophageal cancers over recent decades [3]. Numerous lifestyle-related risk factors have been identified as contributors to the development of these malignancies, each predominantly associated with a specific site of origin—whether esophageal, cardia, or non-cardia gastric cancer—or histological subtype. Notably, regional variations in the incidence of gastric cancer can largely be attributed to environmental influences, as demonstrated by multiple migrant studies [4]. Existing literature largely agrees that EO and late-onset (LO) gastroesophageal tumors exhibit substantial clinicopathological differences, irrespective of geographical region [5–10]. In gastric cancer specifically, EO cases are more likely to occur in women, present at a more advanced stage, be of higher grade, and include a greater proportion of diffuse and signet ring cell carcinomas (SRCC), as well as distinct molecular profiles [11, 12].

The objective of this study is to assess whether these patterns are also observed in the Czech population, thereby confirming or challenging findings reported in other regions, and to evaluate key real-world components of treatment, including participation in clinical trials and the choice of treatment regimens. According to the European Society for Medical Oncology (ESMO), AYAs are categorized as individuals aged 20–39 years [13]. However, the age thresholds used to define EO cancers vary widely in the broader literature, typically ranging from 39 to 49 years [6–8, 14–16]. In this study, patients aged 49 years or younger are classified as having EO disease.

## Methods

### Study design and patients

This retrospective study analyzed a consecutive cohort of adult patients (≥ 18 years) with histologically confirmed esophageal, gastroesophageal junction (GEJ), or primary gastric carcinoma. Data was obtained from the electronic healthcare database of Masaryk Memorial Cancer Institute (MMCI). The study was approved by the Institutional Ethical Committee of MMCI (approval No. 2024/179/MOU), and all participants provided informed consent for the use of their data for research purposes.

Patient diagnoses were determined using ICD-10 codes (International Classification of Diseases, 10th revision) from outpatient and/or inpatient records. The study included adult patients with at least one documented diagnosis of gastroesophageal cancers (ICD-10: C15-C16) between 2010 and 2022. Patients with missing histological confirmation, non-epithelial tumors (e.g., sarcomas, neuroendocrine neoplasms), non-gastroesophageal primary origin, or diagnoses outside the study period were excluded.

**Fig. 1:**
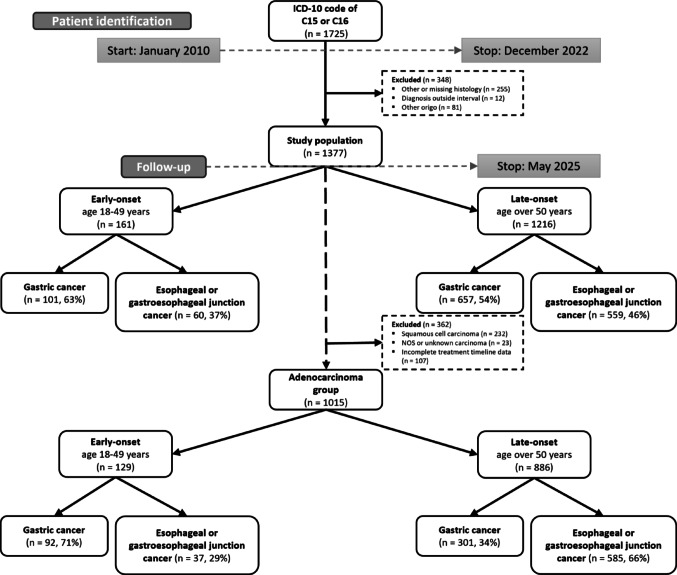
Flowchart of the study population and adenocarcinoma group. Abbreviations: ICD, International Classification of Diseases; NOS, not otherwise specified

For the study, two cohorts were established. The first cohort included all eligible patients and was used to describe the clinicopathological characteristics. The second cohort, used to evaluate treatment outcomes, consisted exclusively of adenocarcinoma patients with complete treatment timeline data. Patient selection and cohort assignment are illustrated in the flowchart (Fig. 1).

Collected data included sex, race, date of diagnosis and last visit of follow-up or death, clinical characteristic at diagnosis (age, Eastern Cooperative Oncology Group (ECOG) performance status, body mass index (BMI), smoking status), primary site, disease stage, metastasis sites (in EO patients only), tumor, pathological and molecular characteristics, clinical trial enrollment, genetic examination, and treatment details (date of surgery and systemic anti-cancer therapy).

All diagnostic procedures, pathological analysis, and treatment were conducted by the established standards of care within our institution and in alignment with the relevant international guidelines. Surgical data were not collected in a fully standardized manner across the entire study period. In routine clinical practice, esophageal cancers were primarily treated with transthoracic esophagectomy, most commonly using McKeown or Ivor Lewis approaches. Gastric cancers were managed with subtotal or total gastrectomy combined with D2 lymphadenectomy, depending on tumor location and extent. The choice of surgical approach was individualized based on tumor characteristics, patient fitness, and surgeon discretion. Systemic treatment dose modifications were performed at the discretion of the treating physician, in accordance with guideline recommendations and individual patient condition. Histological subtypes were classified according to the 4th (2010) and 5th (2019) editions of the WHO classification of digestive tumors [17]. All predictive biomarkers were examined using standardized immunohistochemistry methods. HER-2 examination was conducted throughout the entire period; other biomarkers (MMR status, Epstein-Barr virus (EBV), and PD-L1 positivity) were examined starting in 2014.

Treatment lines were determined according to the methodology proposed by Saini and Twelves [18]. Treatment regimens were categorized based on the number of cytotoxic agents and the presence of immunotherapy or targeted therapy. Regimens containing a single anti-cancer agent were classified as monotherapy. Regimens combining two cytotoxic agents were categorized as doublets, while those with three cytotoxic agents were classified as triplets.

### Long-term outcomes and statistical analysis

Overall survival (OS) was defined as the time from diagnosis to death from any cause. Disease-free survival (DFS) was defined as the time from the date of surgery to the first relapse or death from any cause in patients who underwent radical resection. Patients without an observed event or those lost to follow-up were censored at the date of the last visit.

Patient and treatment characteristics were summarized using standard descriptive statistics. Continuous variables were summarized as medians with interquartile ranges (IQRs), while categorical variables were presented as counts and percentages. Depending on the nature of the data, Fisher’s exact test or the chi-square test was used for categorical variables, and the nonparametric Mann–Whitney test was used for continuous variables to compare groups. Multiple testing corrections were performed using the Holm method. Survival outcomes were estimated using the Kaplan–Meier method and compared with the log-rank test. The univariable and multivariable analyses were conducted in the adenocarcinoma subgroup and separately in patients with inoperable or metastatic adenocarcinoma using the Cox proportional hazards model, and effects were expressed as hazard ratios (HR). Variables for the multivariable model were selected using a stepwise approach. Follow-up time was determined using the reverse Kaplan–Meier method. Median survival times and survival rates were reported with 95% confidence intervals (CIs). *P*-values of < 0.05 were considered statistically significant. All statistical analyses were performed in R, version 4.5.0.

## Results

### Cohort characteristics

A study was conducted on a cohort of 1,377 patients diagnosed with gastroesophageal carcinoma, predominantly White individuals (6 patients of other racial backgrounds). The median age at diagnosis was 64 years (range, 20–94). The analysis compared 161 patients with EO disease and 1,216 patients with LO disease.

In both age groups, males were more commonly affected; however, male predominance was less pronounced in the EO group (63% vs. 72% in the LO group, *p* = 0.013). Interestingly, among EO patients with gastric cancer, the sex distribution was nearly balanced (53% male and 47% female). Most patients in both groups had a good performance status (ECOG PS 0–1), and EO patients were more frequently classified as having a performance status of 0 (*p* < 0.001).

### Risk factors

The proportion of never-smokers was similar in both groups (34% in the EO group vs. 37% in the LO group). However, current smoking was more common among EO patients (44% vs. 27%; *p* < 0.001), especially among those with gastric cancer (48% vs. 21%). LO patients had a higher median BMI of 25.1 compared to 23.9 (*p* = 0.005) and were more often classified as obese (13% vs. 7.3%). The highest obesity rate (20%) was observed in the LO GEJ cancer subgroup. Patients’ characteristics are summarized in Table 1 and Supplementary Table 1.

**Table 1:** Clinical and pathological characteristics of patients in the study population

	Early-onset *N* = 161	Late-onset *N* = 1,216	*p*-value
*Year of diagnosis*			0.639
2010–2012	38 (24%)	324 (27%)	
2013–2015	34 (21%)	281 (23%)	
2016–2018	51 (32%)	334 (27%)	
2019–2021	38 (24%)	277 (23%)	
*ECOG performance status*			**0.006**
0	62 (42%)	331 (29%)	
1	58 (40%)	608 (53%)	
2	19 (13%)	144 (13%)	
3–4	7 (4.8%)	59 (5.2%)	
Missing	15	74	
*Sex*			**0.013**
Male	101 (63%)	878 (72%)	
Female	60 (37%)	338 (28%)	
*Age (years)*			**< 0.001**
Median (IQR)	44 (40, 47)	66 (60, 72)	
Range	20, 49	50, 94	
*Smokers*			**< 0.001**
Never	49 (34%)	423 (37%)	
Former	33 (23%)	412 (36%)	
Current	64 (44%)	316 (27%)	
Missing	15	65	
*Body mass index (BMI)*			**0.005**
Median (IQR)	23.9 (20.3, 27.0)	25.1 (22.2, 28.2)	
Range	15.3, 43.0	13.3, 46.1	
Underweight	12 (11%)	66 (7.3%)	0.085
Normal weight	55 (50%)	384 (42%)	
Overweight	35 (32%)	334 (37%)	
Obesity	8 (7.3%)	122 (13%)	
Missing	51	310	
*Primary site*			**0.006**
Esophageal	30 (19%)	374 (31%)	
GEJ	30 (19%)	185 (15%)	
Gastric	101 (63%)	657 (54%)	
*Histology*			**0.004**
Adenocarcinoma	145 (91%)	977 (80%)	
Squamous cell carcinoma	15 (9.4%)	217 (18%)	
NOS carcinoma	0 (0%)	20 (1.6%)	
Unknown	1	2	
*Adenocarcinoma subtype*			**< 0.001**
Poorly cohesive	65/145 (45%)	223/957 (23%)	
Tubular	15/145 (10%)	45/957 (4.7%)	
Mucinous	9/145 (6.2%)	25/957 (2.6%)	
Other	13/145 (9.0%)	19/957 (2.0%)	
NS	43/145 (30%)	645/957 (67%)	
Unknown	0	20	
*Stage at diagnosis*			**0.003**
Localized	6 (3.8%)	95 (7.9%)	
Locally advanced	66 (41%)	610 (51%)	
Metastatic	88 (55%)	497 (41%)	
Unknown	1	14	
**Visceral involvement**	37/86 (43%)		
**LAP**	21/86 (24%)		
**Peritoneal metastases**	48/86 (56%)		
**Bone metastases**	6/86 (7.0%)		
**Other metastatic sites**	18/86 (21%)		
**HER2 positive** ^*^	16/84 (19%)	53/404 (13%)	0.156
**dMMR**	2/34 (5.9%)	10/136 (7.4%)	> 0.999
**EBV positive**	0/7 (0%)	2/50 (4.0%)	> 0.999
**PD-L1 positive** ^**+**^	9/28 (32%)	42/123 (34%)	0.840
**Treated in clinical trials**	20 (12%)	121 (10.0%)	0.331
**Genetic counseling**	32 (20%)	30 (2.5%)	**< 0.001**
*Genetic results*			0.833
No mutation	25/32 (78%)	26/30 (87%)	
GAPPS	4/32 (13%)	2/30 (6.7%)	
Hereditary diffuse gastric cancer	1/32 (3.1%)	0/30 (0%)	
Lynch syndrome	1/32 (3.1%)	1/30 (3.3%)	
Hereditary breast and ovarian cancer syndrome	0/32 (0%)	1/30 (3.3%)	
Familial melanoma syndrome	1/32 (3.1%)	0/30 (0%)	

* HER-2 positivity is defined as IHC 3 + or IHC 2 + with ISH confirmed amplification

+ PD-L1 positivity is defined as follows: for adenocarcinoma, CPS ≥ 5; for squamous histology, TPS ≥ 1%

Bold values indicate statistical significance (*p* < 0.05)

Abbreviations: dMMR, mismatch repair deficient; EBV, Epstein-Barr virus; ECOG, Eastern Cooperative Oncology Group; GAPPS, gastric adenocarcinoma and proximal polyposis of the stomach; GEJ, gastroesophageal junction; HER-2, human epidermal growth factor receptor 2; IQR, interquartile range; LAP, lymphadenopathy; NOS, not otherwise specified; NS, not specified, PD-L1, programmed-death ligand 1

**Table 2:** Univariable analysis for overall survival in the adenocarcinoma subgroup

	All adenocarcinomas				Inoperable/Metastatic			
	*N*	Number of deaths	HR (95% CI)	*p*-value	*N*	Number of deaths	HR (95% CI)	*p*-value
*Group*				0.253				0.912
Early-onset	129	110	–		93	89	–	
Late-onset	886	733	0.89 (0.73, 1.09)		529	511	0.99 (0.79, 1.24)	
*ECOG performance status*				< 0.001*				< 0.001*
0	318	238	–		143	137	–	
1	510	431	1.42 (1.21, 1.67)		328	316	1.14 (0.93, 1.39)	
2	116	108	2.72 (2.16, 3.42)		96	92	1.77 (1.36, 2.31)	
3–4	56	53	3.89 (2.89, 5.25)		45	45	7.93 (5.57, 11.3)	
*Sex*				0.031*				0.181
Male	697	594	–		432	420	–	
Female	318	249	0.85 (0.73, 0.99)		190	180	0.89 (0.75, 1.06)	
*Age (years)*	1,015	843	1.00 (1.00, 1.01)	0.573	622	600	1.00 (1.00, 1.01)	0.340
*Smokers*				0.573				0.766
Never	392	319	–		247	236	–	
Former	323	269	1.07 (0.91, 1.26)		189	184	1.07 (0.88, 1.29)	
Current	257	219	1.09 (0.92, 1.29)		158	153	1.06 (0.86, 1.30)	
*Body mass index*				0.072				0.321
Normal weight	338	294	–		225	220	–	
Underweight	37	32	1.03 (0.71, 1.48)		27	27	1.05 (0.71, 1.57)	
Overweight	312	248	0.81 (0.68, 0.96)		182	171	0.85 (0.70, 1.04)	
Obesity	110	88	0.85 (0.67, 1.08)		71	68	0.84 (0.64, 1.11)	
*Primary site*				**0.018**				> 0.999
Esophageal/GEJ	338	292	–		218	212	–	
Gastric	677	551	0.84 (0.73, 0.97)		404	388	1.00 (0.85, 1.18)	
*Adenocarcinoma subtype*				0.046*				0.436
Poorly cohesive	263	227	–		164	160	–	
Other	736	601	0.85 (0.73, 1.00)		446	428	0.93 (0.77, 1.12)	
*Stage disease*				< 0.001*				
Localized/Locally advanced, radically resected	393	243	–					
Inoperable/Metastatic	622	600	4.63 (3.94, 5.44)					

*Remained significant in the multivariable analysis

Bold values indicate statistical significance (*p* < 0.05)

Abbreviations: CI, confidence interval; ECOG, Eastern Cooperative Oncology Group; GEJ, gastroesophageal junction; HR, hazard ratio

### Primary site and histology

The distribution of tumor sites and histologic subtypes differed significantly between EO and LO patients (*p* = 0.006 and *p* = 0.004, respectively). Primary gastric cancer was the most common tumor site overall (55%) and was more frequent in EO patients (63% vs. 54%). GEJ cancer was also slightly more common in the EO group (19% vs. 15%), whereas esophageal cancer was more commonly diagnosed in LO patients (31% vs. 19%).

Adenocarcinoma was the predominant histologic subtype in both groups (91% in EO vs. 80% in LO). Poorly cohesive adenocarcinoma was the most frequent subtype in EO patients (45% vs. 23%), followed by tubular (10% in EO vs. 4.7%), mucinous (6.2% in EO vs. 2.6%), and other types (9% in EO vs. 2.0%). In contrast, LO adenocarcinomas were classified as non-specified (67% vs. 30%). Squamous cell carcinoma was more common in LO patients (18% vs. 9.4%).

HER2 status was available in 488 patients. A higher, though statistically insignificant, HER2 positivity was observed in EO patients (19% vs. 13%; *p* = 0.156). All predictive biomarker assessments are reported in Table 1 and Supplementary Table 1.

### Staging

EO patients were more frequently diagnosed with metastatic disease (55% vs. 41%; *p* = 0.001). Among EO patients with metastatic disease, peritoneal metastases were most common (56%), followed by visceral involvement (43%), non-regional lymph node infiltration (24%), bone metastases (7%), and other metastatic sites (21%). Among female patients, primary metastatic disease was significantly more common in the EO group than in LO females (65% vs. 44%; *p* = 0.003). Within the EO group, females also had a higher rate of more advanced stages than males (65% vs. 49%, *p* = 0.049), whose rate of stage IV disease was comparable to that of LO males (40%).

### Genetic examination

Genetic counseling and germline testing were performed significantly more often in EO patients (20% vs. 2.5%; *p* < 0.001). A hereditary cancer syndrome was identified in 7 patients (22% of 32 tested EO patients), most frequently gastric adenocarcinoma and proximal polyposis of the stomach (GAPPS; 4 patients), followed by one case each of hereditary diffuse gastric cancer, Lynch syndrome, and familial melanoma syndrome. In the LO group, 87% of 30 tested patients had no detected pathogenic mutation; GAPPS was found in 2 cases, and Lynch syndrome and hereditary breast and ovarian cancer syndrome were each identified in 1 patient.

### Treatment outcomes in the adenocarcinoma subgroup

**Fig. 2:**
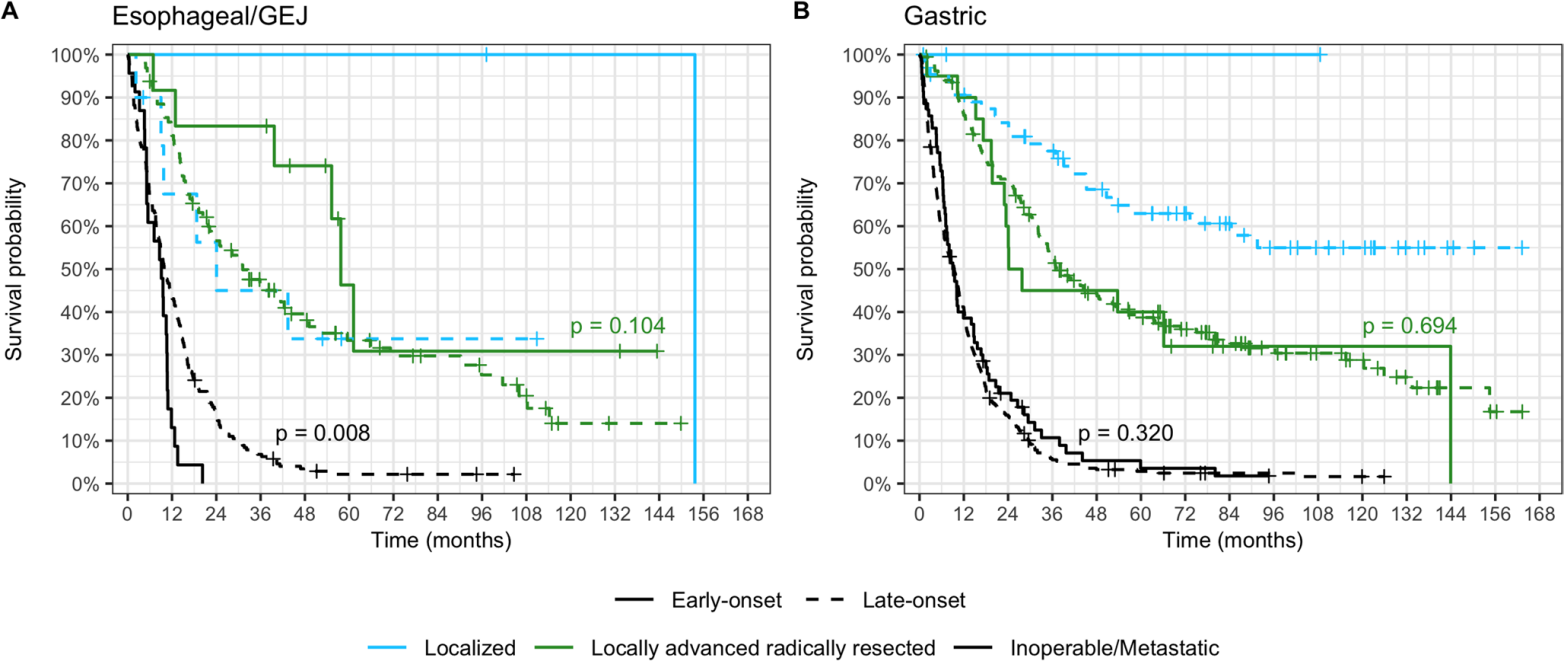
Overall survival in the adenocarcinoma subgroup stratified by disease localization and age. Solid lines represent EO patients and dashed lines represent LO patients; disease extent is indicated by colors. Abbreviations: GEJ, gastroesophageal junction

The median follow-up period for the 1,015 patients in the adenocarcinoma subgroup, which included 129 patients with EO and 886 patients with LO cancer, was 89.1 months (95% CI: 80.8–99.4). Median OS was 36.9 months (95% CI: 32.6–48.1) for patients with locally advanced, operable disease, while it was 9.5 months (95% CI: 8.4–10.3) for those with inoperable or metastatic disease (see Fig. 2). A statistically significant difference was noted between EO and LO patients only among those with inoperable or metastatic esophageal or GEJ cancer (*p* = 0.008). Baseline characteristics of patients in the adenocarcinoma subgroup are summarized in Supplementary Tables 2, and detailed OS parameters can be found in Supplementary Table 3.

In the overall adenocarcinoma subgroup (Table 2, Supplementary Table 4), worse performance status, male sex, esophageal or GEJ cancer, and inoperable or metastatic disease were associated with poorer OS. Among patients with inoperable or metastatic adenocarcinoma (Table 2, Supplementary Table 4), performance status remained the sole significant factor. In esophageal or GEJ adenocarcinoma (Supplementary Tables 5 and 6), performance status and disease stage retained statistical significance. Conversely, in inoperable or metastatic gastric adenocarcinoma (Supplementary Tables 7 and 8), sex, performance status, and adenocarcinoma subtype were identified as independent prognostic factors.

Only 32 EO patients were diagnosed with locally advanced operable cancer, with a median DFS of 18.6 months. Neoadjuvant or perioperative therapy was administered to 15 (47%) of the EO patients and 116 (41%) of the LO patients, with no significant difference between the two groups (*p* = 0.543). Of the 93 EO and 529 LO patients with locally advanced inoperable or metastatic adenocarcinoma, systemic anti-cancer therapy was initiated in 85% and 74%, respectively (*p* = 0.020). In the first line setting, EO patients more often received triplet chemotherapy regimens (29% vs. 11%; *p* < 0.001). Regarding specific agents used, EO patients more frequently received docetaxel-based (20% vs. 8.4%; *p* = 0.021) and anthracycline-based (13% vs. 4.3%; *p* = 0.056) combinations. Nearly all EO patients (99%) were treated with a platinum-based regimen, compared to 89% of LO patients (*p* = 0.075). EO patients were more likely to receive subsequent lines of therapy, although the difference was not statistically significant. Detailed treatment characteristics are summarized in Table 3.

**Table 3:** Treatment strategies and systemic treatment regimens in the adenocarcinoma subgroup

	Early-onset	Late-onset	*p*-value	Adjusted *p*-value*
**Primary tumor surgery in patients with locally advanced disease at diagnosis**			**<0.001**	
Radical	32/50 (64%)	281/424 (66%)		
Non-radical	12/50 (24%)	27/424 (6%)		
None	6/50 (12%)	116/424 (27%)		
**Treatment strategy (Locally advanced, radically resected)**			0.376	
Neoadjuvant therapy	7 (22%)	61 (22%)		
Perioperative therapy	8 (25%)	55 (20%)		
Adjuvant therapy	15 (47%)	114 (41%)		
Surgery alone	2 (6.3%)	51 (18%)		
**BSC only**	14/93 (15%)	139/529 (26%)	**0.020**	
**BSC-only duration (weeks)**	12 (2, 46)	11 (0, 257)	0.541	
Unknown	0	1		
**Systemic therapy duration (weeks)**	26 (0, 334)	29 (0, 431)	0.701	
Unknown	4	4		
**BSC duration after systemic therapy (weeks)**	9 (1, 65)	10 (0, 260)	0.065	
Unknown	8	20		
**First-line treatment**	79/93 (85%)	390/529 (74%)	**0.020**	
Monotherapy	1/79 (1.3%)	29/389 (7.5%)	**0.041**	0.122
Doublet	45/79 (57%)	273/389 (70%)	**0.022**	0.087
Triplet	23/79 (29%)	43/389 (11%)	** < 0.001**	** < 0.001**
Other	0/79 (0%)	1/389 (0.3%)	> 0.999	> 0.999
Trial	10/79 (13%)	43/389 (11%)	0.682	> 0.999
Platinum-based	68/69 (99%)	309/346 (89%)	**0.015**	0.075
Docetaxel-based	14/69 (20%)	29/346 (8.4%)	**0.003**	**0.021**
Paclitaxel-based	0/69 (0%)	17/346 (4.9%)	0.089	0.355
Irinotecan-based	1/69 (1.4%)	5/346 (1.4%)	> 0.999	> 0.999
Anthracycline-based	9/69 (13%)	15/346 (4.3%)	**0.009**	0.056
Immunotherapy^‡^	0/69 (0%)	1/346 (0.3%)	> 0.999	> 0.999
Targeted therapy^#^	7/69 (10%)	22/346 (6.4%)	0.298	0.893
**First-line treatment duration (weeks)**	16 (0, 150)	15 (0, 104)	0.996	
**Second-line treatment**	48/76 (63%)	209/388 (54%)	0.136	
Unknown	3	2		
Monotherapy	14/48 (29%)	57/209 (27%)	0.791	> 0.999
Doublet	20/48 (42%)	110/209 (53%)	0.171	0.853
Triplet	2/48 (4.2%)	5/209 (2.4%)	0.618	> 0.999
Other	6/48 (13%)	14/209 (6.7%)	0.227	0.908
Trial	6/48 (13%)	23/209 (11%)	0.768	> 0.999
Platinum-based	12/42 (29%)	40/186 (22%)	0.324	0.840
Docetaxel-based	2/42 (4.8%)	9/186 (4.8%)	> 0.999	> 0.999
Paclitaxel-based	14/42 (33%)	44/186 (24%)	0.193	0.773
Irinotecan-based	12/42 (29%)	92/186 (49%)	**0.014**	0.099
Anthracycline-based	2/42 (4.8%)	1/186 (0.5%)	0.088	0.440
Immunotherapy^‡^	2/42 (4.8%)	0/186 (0%)	**0.033**	0.200
Targeted therapy^#^	7/42 (17%)	19/186 (10%)	0.280	0.840
**Second-line treatment duration (weeks)**	9 (0, 156)	10 (0, 114)	0.486	
**Third-line treatment**	25/47 (53%)	83/207 (40%)	0.101	
Unknown	1	2		
**Fourth-line treatment**	6/25 (24%)	22/83 (27%)	0.802	
**Fifth-line treatment**	1/6 (17%)	4/22 (18%)	> 0.999	
**Sixth-line treatment**	0/1 (0%)	1/4 (25%)	> 0.999	

*Holm correction for multiple testing

Categories of treatment are not mutually exclusive

^‡^Regimens containing nivolumab or pembrolizumab

^*#*^Regimens containing trastuzumab (including trastuzumab deruxtecan) or ramucirumab

Bold values indicate statistical significance (*p *< 0.05)

Abbreviations: BSC, best supportive care (defined as the absence of systemic anticancer therapy)

## Discussion

This study represents the first population-based, real-world analysis of gastroesophageal carcinoma in the Czech Republic, encompassing approximately 5% of all national cases and 10% of the EO gastroesophageal cancer population [3]. EO disease accounted for 11.7% of all gastroesophageal cancers, with EO gastric cancer representing 7.7%, a proportion consistent with other European reports [7, 9]. In contrast, studies from the United States describe substantially higher rates of EOGC—exceeding 30%—a difference likely attributable to greater ethnic and environmental heterogeneity and distinct genetic or epigenetic risk profiles [2, 7, 19, 20]. In the United States, Hispanic and Black patients predominate among EOGC cases—accounting for nearly one quarter and representing the groups with the poorest outcomes, respectively [7, 19]—whereas the present study population consisted almost exclusively of individuals identified as White. This composition mirrors the generally homogeneous population structure of the Czech Republic; however, given the retrospective single-center design, the results should be interpreted as descriptive rather than population-based epidemiological estimates. Early-onset gastroesophageal cancer in this cohort was characterized by a predominance of poorly cohesive adenocarcinoma and a high frequency of metastatic disease at diagnosis. These findings are consistent with a large meta-analysis reporting similar histological patterns and a slight female predominance [19].

Germline cancer predisposition syndromes were identified in 4.3% of EO cases. Notably, gastric adenocarcinoma and proximal polyposis of the stomach (GAPPS) was the most frequent syndrome identified, surpassing hereditary diffuse gastric cancer (HDGC), which is typically considered the predominant hereditary entity in this age group [7, 21]. The higher frequency of GAPPS observed in our cohort may be explained by regional family aggregation in South Moravia and referral patterns to our center rather than the nationwide distribution of hereditary gastric cancer syndromes in the Czech Republic [21].

HER2 overexpression was observed more frequently among EO patients, although HER2 testing was also performed more often in this group. This observation is consistent with findings from the NIU HER2 Study Group [22]. These data highlight the clinical relevance of HER2 assessment in younger patients, particularly in the context of evolving perioperative and metastatic anti-HER2 treatment strategies [23, 24]. No significant age-related differences in other biomarker distribution were observed.

Despite publication of the MAGIC trial prior to the study period, nearly half of patients with locally advanced adenocarcinoma were treated with upfront surgery with or without adjuvant chemoradiotherapy rather than guideline-recommended perioperative chemotherapy [25–28]. This treatment pattern likely reflects the period from which we recruited data, urgent surgical presentation, clinical understaging, or initial management in non-specialized centers.

Data on outcomes in inoperable or metastatic EO gastroesophageal cancer remain heterogeneous. While some studies report comparable outcomes between EO and LO patients [29], others describe improved survival in younger individuals with esophageal or gastroesophageal junction adenocarcinoma [6]. In contrast, EO patients with inoperable or metastatic disease in our cohort experienced inferior survival outcomes, with overall survival shorter across all groups compared with previous reports. These discrepancies may be explained by differences in study populations, treatment eras, therapeutic strategies, and baseline disease burden.

Treatment intensity remains an important consideration in younger patients, who typically present with fewer comorbidities and better performance status. In our cohort, EO patients more frequently had ECOG performance status 0 and were more often treated with triplet chemotherapy regimens. Nevertheless, this intensified approach did not translate into a survival advantage. These findings are in line with several retrospective cohorts from Spain and Japan, which demonstrated comparable efficacy and toxicity across age groups despite more intensive treatment in younger patients [29, 30]. Conversely, the prospective GASTFOX trial reported improved survival with triplet TFOX chemotherapy in patients without actionable molecular targets, with a more pronounced benefit observed in younger adults in subgroup analyses [31]. Together, these conflicting results underscore the need for prospective, EOGC-focused trials to define optimal treatment intensity. Importantly, participation in clinical trials was comparable between EO and LO patients in our cohort.

Although comorbidity data could not be comprehensively extracted, treatment decisions in our cohort were made in the context of real-world multidisciplinary evaluation, reflecting clinical judgment that integrates performance status, frailty, and patient preference. As expected, younger patients presented with lower comorbidity burden, indirectly reflected by their higher proportion of ECOG 0. In older or frail patients, dose reduction of fluoropyrimidine-based therapy—as supported by the GO2 trial—remains a rational strategy to balance efficacy and tolerability [32].

Several limitations warrant consideration. The retrospective, single-center design introduces potential selection and reporting biases and may limit generalizability, particularly given our role as a national referral center for younger and more advanced cases. Key risk factors—including alcohol consumption, gastroesophageal reflux disease, family history, and *Helicobacter pylori* infection—were inconsistently documented, precluding comprehensive risk assessment [12, 19]. Diagnostic delay, a critical prognostic factor in adolescent and young adult oncology, could not be reliably evaluated due to incomplete data from referring institutions.

In conclusion, this Central European real-world analysis demonstrates that EO gastroesophageal cancer is characterized by distinct clinicopathological features, including a predominance of poorly cohesive histology and advanced-stage presentation. Although more intensive chemotherapy regimens were frequently used in younger patients, no associated survival benefit was observed. These findings highlight the importance of centralized care, systematic molecular and genetic evaluation, and prospective outcome monitoring to optimize management strategies for early-onset gastroesophageal cancer.

## Electronic Supplementary Material

Below is the link to the electronic supplementary material.

Supplementary Material 1

## Abbreviations

dMMR: Mismatch repair deficient
EBV: Epstein-Barr virus
ECOG: Eastern Cooperative Oncology Group
GAPPS: Gastric adenocarcinoma and proximal polyposis of the stomach
GEJ: Gastroesophageal junction
HER-2: Human epidermal growth factor receptor 2
IQR: Interquartile range
LAP: Lymphadenopathy
NOS: Not otherwise specified
NS: Not specified, PD-L1,programmed-death ligand 1

## Declarations

### Human and animal participants

All procedures followed were in accordance with the ethical standards of the responsible committee on human experimentation (institutional and national) and with the Helsinki Declaration of 1964 and later versions.
