# Supplementary material for: Early- versus late-onset gastroesophageal cancer: real-world outcomes from a 13-year central European cohort study
Source: Gastric Cancer. 2026 Mar 27;29(3):494–505. doi: 10.1007/s10120-026-01724-z (PMC13124764; doi:10.1007/s10120-026-01724-z)
Supplement: Supplementary file 1 — Supplementary Material 1 [file 10120_2026_1724_MOESM1_ESM.docx]

**Supplementary Table 1.** Clinical and pathological characteristics of patients in the study population stratified by disease localization and age.

|  | **Esophageal** | | | **GEJ** | | | **Gastric** | | |
| --- | --- | --- | --- | --- | --- | --- | --- | --- | --- |
|  | **Early-onset**  N = 30 | **Late-onset**  N = 374 | **p-value** | **Early-onset**  N = 30 | **Late-onset**  N = 185 | **p-value** | **Early-onset**  N = 101 | **Late-onset**  N = 657 | **p-value** |
| **Year of diagnosis** |  |  | 0.890 |  |  | 0.389 |  |  | 0.122 |
| 2010-2012 | 7 (23%) | 77 (21%) |  | 8 (27%) | 28 (15%) |  | 23 (23%) | 219 (33%) |  |
| 2013-2015 | 6 (20%) | 99 (26%) |  | 7 (23%) | 39 (21%) |  | 21 (21%) | 143 (22%) |  |
| 2016-2018 | 8 (27%) | 95 (25%) |  | 7 (23%) | 61 (33%) |  | 36 (36%) | 178 (27%) |  |
| 2019-2021 | 9 (30%) | 103 (28%) |  | 8 (27%) | 57 (31%) |  | 21 (21%) | 117 (18%) |  |
| **ECOG performance status** |  |  | 0.501 |  |  | 0.160 |  |  | **0.022** |
| 0 | 5 (19%) | 84 (24%) |  | 13 (48%) | 51 (30%) |  | 44 (47%) | 196 (32%) |  |
| 1 | 15 (58%) | 205 (58%) |  | 9 (33%) | 92 (54%) |  | 34 (37%) | 311 (50%) |  |
| 2 | 6 (23%) | 49 (14%) |  | 4 (15%) | 18 (11%) |  | 9 (9.7%) | 77 (12%) |  |
| 3-4 | 0 (0%) | 16 (4.5%) |  | 1 (3.7%) | 9 (5.3%) |  | 6 (6.5%) | 34 (5.5%) |  |
| Missing | 4 | 20 |  | 3 | 15 |  | 8 | 39 |  |
| **Sex** |  |  | 0.782 |  |  | **0.032** |  |  | 0.140 |
| Male | 27 (90%) | 322 (86%) |  | 20 (67%) | 154 (83%) |  | 54 (53%) | 402 (61%) |  |
| Female | 3 (10%) | 52 (14%) |  | 10 (33%) | 31 (17%) |  | 47 (47%) | 255 (39%) |  |
| **Age (years)** |  |  | **<0.001** |  |  | **<0.001** |  |  | **<0.001** |
| Median (IQR) | 45 (41, 47) | 65 (60, 70) |  | 45 (42, 47) | 67 (60, 73) |  | 43 (39, 47) | 66 (60, 72) |  |
| Range | 30, 49 | 50, 87 |  | 23, 49 | 50, 90 |  | 20, 49 | 50, 94 |  |
| **Smokers** |  |  | 0.245 |  |  | 0.276 |  |  | **<0.001** |
| Never | 9 (32%) | 69 (19%) |  | 10 (38%) | 66 (38%) |  | 30 (33%) | 288 (47%) |  |
| Former | 9 (32%) | 149 (41%) |  | 6 (23%) | 63 (36%) |  | 18 (20%) | 200 (32%) |  |
| Current | 10 (36%) | 142 (39%) |  | 10 (38%) | 44 (25%) |  | 44 (48%) | 130 (21%) |  |
| Missing | 2 | 14 |  | 4 | 12 |  | 9 | 39 |  |
| **Body mass index (BMI)** |  |  | 0.359 |  |  | 0.150 |  |  | **0.005** |
| Median (IQR) | 23.4 (19.8, 26.9) | 24.3 (20.9, 28.2) |  | 25.3 (21.4, 27.8) | 26.7 (23.5, 29.4) |  | 23.6 (19.7, 26.8) | 25.1 (22.5, 27.6) |  |
| Range | 15.3, 31.7 | 13.3, 40.1 |  | 19.9, 30.5 | 15.0, 43.6 |  | 16.3, 43.0 | 13.3, 46.1 |  |
| Underweight | 1 (5.6%) | 41 (14%) | 0.534 | 0 (0%) | 5 (3.8%) | 0.330 | 11 (15%) | 20 (4.2%) | **0.003** |
| Normal weight | 10 (56%) | 122 (42%) |  | 9 (47%) | 43 (32%) |  | 36 (49%) | 219 (46%) |  |
| Overweight | 6 (33%) | 88 (30%) |  | 9 (47%) | 58 (44%) |  | 20 (27%) | 188 (39%) |  |
| Obesity | 1 (5.6%) | 41 (14%) |  | 1 (5.3%) | 27 (20%) |  | 6 (8.2%) | 54 (11%) |  |
| Missing | 12 | 82 |  | 11 | 52 |  | 28 | 176 |  |
| **Histology** |  |  | 0.642 |  |  | >0.999 |  |  | 0.338 |
| Adenocarcinoma | 15 (52%) | 157 (42%) |  | 29 (97%) | 178 (97%) |  | 101 (100%) | 642 (98%) |  |
| Squamous cell carcinoma | 14 (48%) | 210 (56%) |  | 1 (3.3%) | 6 (3.3%) |  | 0 (0%) | 1 (0.2%) |  |
| NOS carcinoma | 0 (0%) | 6 (1.6%) |  | 0 (0%) | 0 (0%) |  | 0 (0%) | 14 (2.1%) |  |
| Unknown | 1 | 1 |  | 0 | 1 |  |  |  |  |
| **Adenocarcinoma subtype** |  |  | **0.032** |  |  | **<0.001** |  |  | **<0.001** |
| Poorly cohesive | 0 (0%) | 9 (5.9%) |  | 7 (24%) | 23 (13%) |  | 58 (57%) | 191 (30%) |  |
| Tubular | 0 (0%) | 2 (1.3%) |  | 2 (6.9%) | 5 (2.9%) |  | 13 (13%) | 38 (6.0%) |  |
| Mucinous | 0 (0%) | 3 (2.0%) |  | 3 (10%) | 4 (2.3%) |  | 6 (5.9%) | 18 (2.8%) |  |
| Other | 2 (13%) | 0 (0%) |  | 3 (10%) | 2 (1.2%) |  | 8 (7.9%) | 17 (2.7%) |  |
| NS | 13 (87%) | 139 (91%) |  | 14 (48%) | 138 (80%) |  | 16 (16%) | 368 (58%) |  |
| Unknown | 0 | 4 |  | 0 | 6 |  | 0 | 10 |  |
| **Stage at diagnosis** |  |  | 0.302 |  |  | 0.791 |  |  | **0.004** |
| Localized | 1 (3.4%) | 17 (4.6%) |  | 1 (3.3%) | 4 (2.2%) |  | 4 (4.0%) | 74 (11%) |  |
| Locally advanced | 14 (48%) | 227 (62%) |  | 17 (57%) | 103 (56%) |  | 35 (35%) | 280 (43%) |  |
| Metastatic | 14 (48%) | 125 (34%) |  | 12 (40%) | 77 (42%) |  | 62 (61%) | 295 (45%) |  |
| Missing | 1 | 5 |  | 0 | 1 |  | 0 |  |  |
| **Visceral involvement** | 9/13 (69%) |  |  | 6/12 (50%) |  |  | 22/61 (36%) |  |  |
| **LAP** | 6/13 (46%) |  |  | 5/12 (42%) |  |  | 10/61 (16%) |  |  |
| **Peritoneal metastases** | 0/13 (0%) |  |  | 4/12 (33%) |  |  | 44/61 (72%) |  |  |
| **Bone metastases** | 0/13 (0%) |  |  | 1/12 (8.3%) |  |  | 5/61 (8.2%) |  |  |
| **Other metastatic sites** | 3/13 (23%) |  |  | 3/12 (25%) |  |  | 12/61 (20%) |  |  |
| **HER2 positive^*^** | 4/7 (57%) | 7/46 (15%) | **0.027** | 3/13 (23%) | 10/69 (14%) | 0.424 | 9/64 (14%) | 36/289 (12%) | 0.727 |
| **dMMR** | 0/3 (0%) | 3/27 (11%) |  | 0/10 (0%) | 0/23 (0%) |  | 2/21 (9.5%) | 7/86 (8.1%) |  |
| **EBV positive** | 0/0 | 0/13 (0%) |  | 0/0 | 0/7 (0%) |  | 0/7 (0%) | 2/30 (6.7%) |  |
| **PD-L1 positive^+^** | 4/5 (80%) | 12/32 (38%) | 0.144 | 1/8 (13%) | 7/23 (30%) | 0.642 | 4/15 (27%) | 23/68 (34%) | 0.764 |
| **Treated in clinical trials** | 3 (10%) | 32 (8.6%) | 0.736 | 6 (20%) | 37 (20%) | >0.999 | 11 (11%) | 52 (7.9%) | 0.313 |
| **Genetic examination** | 1 (3.3%) | 1 (0.3%) | 0.143 | 3 (10%) | 1 (0.5%) | **0.009** | 28 (28%) | 28 (4.3%) | **<0.001** |
| **Genetic results** |  |  | >0.999 |  |  | >0.999 |  |  | >0.999 |
| No mutation | 0/1 (0%) | 1/1 (100%) |  | 2/3 (67%) | 1/1 (100%) |  | 23/28 (82%) | 24/28 (86%) |  |
| GAPPS | 0/1 (0%) | 0/1 (0%) |  | 1/3 (33%) | 0/1 (0%) |  | 3/28 (11%) | 2/28 (7.1%) |  |
| Hereditary diffuse gastric cancer | 0/1 (0%) | 0/1 (0%) |  | 0/3 (0%) | 0/1 (0%) |  | 1/28 (3.6%) | 0/28 (0%) |  |
| Lynch syndrome | 1/1 (100%) | 0/1 (0%) |  | 0/3 (0%) | 0/1 (0%) |  | 0/28 (0%) | 1/28 (3.6%) |  |
| Hereditary breast and ovarian cancer syndrome | 0/1 (0%) | 0/1 (0%) |  | 0/3 (0%) | 0/1 (0%) |  | 0/28 (0%) | 1/28 (3.6%) |  |
| Familial melanoma syndrome | 0/1 (0%) | 0/1 (0%) |  | 0/3 (0%) | 0/1 (0%) |  | 1/28 (3.6%) | 0/28 (0%) |  |

* HER-2 positivity is defined as IHC 3+ or IHC 2+ with ISH confirmed amplification

+ PD-L1 positivity is defined as follows: for adenocarcinoma, CPS ≥5; for squamous histology, TPS≥1%

Bold values indicate statistical significance (p < 0.05).

Abbreviations: dMMR, mismatch repair deficient; EBV, Epstein-Barr virus; ECOG, Eeastern Cooperative Oncology Group; GAPPS, gastric adenocarcinoma and proximal polyposis of the stomach; GEJ, gastroesophageal junction; HER-2, human epidermal growth factor receptor 2; IQR, interquartile range; LAP, lymphadenopathy; NOS, not otherwise specified; NS, not specified, PD-L1, programmed-death ligand 1

**Supplementary Table 2.** Clinical and pathological characteristics of patients in the adenocarcinoma group stratified by disease stage and age.

|  | **Localized** | | | **Locally advanced, radically resected** | | | **Inoperable/Metastatic** | | |
| --- | --- | --- | --- | --- | --- | --- | --- | --- | --- |
|  | **Early-onset**  N = 4 | **Late-onset**  N = 76 | **p-value** | **Early-onset**  N = 32 | **Late-onset**  N = 281 | **p-value** | **Early-onset**  N = 93 | **Late-onset**  N = 529 | **p-value** |
| **Year of diagnosis** |  |  | 0.603 |  |  | 0.322 |  |  | 0.501 |
| 2010-2012 | 3 (75%) | 32 (42%) |  | 8 (25%) | 78 (28%) |  | 18 (19%) | 141 (27%) |  |
| 2013-2015 | 0 (0%) | 12 (16%) |  | 6 (19%) | 79 (28%) |  | 20 (22%) | 107 (20%) |  |
| 2016-2018 | 0 (0%) | 18 (24%) |  | 12 (38%) | 65 (23%) |  | 30 (32%) | 160 (30%) |  |
| 2019-2021 | 1 (25%) | 14 (18%) |  | 6 (19%) | 59 (21%) |  | 25 (27%) | 121 (23%) |  |
| **ECOG performance status** |  |  | 0.134 |  |  | 0.053 |  |  | 0.102 |
| 0 | 3 (75%) | 36 (48%) |  | 20 (65%) | 116 (42%) |  | 30 (33%) | 113 (22%) |  |
| 1 | 0 (0%) | 31 (41%) |  | 10 (32%) | 141 (51%) |  | 40 (44%) | 288 (55%) |  |
| 2 | 1 (25%) | 3 (4.0%) |  | 0 (0%) | 16 (5.8%) |  | 14 (16%) | 82 (16%) |  |
| 3-4 | 0 (0%) | 5 (6.7%) |  | 1 (3.2%) | 5 (1.8%) |  | 6 (6.7%) | 39 (7.5%) |  |
| Missing | 0 | 1 |  | 1 | 3 |  | 3 | 7 |  |
| **Sex** |  |  | 0.633 |  |  | 0.074 |  |  | **0.019** |
| Male | 3 (75%) | 43 (57%) |  | 18 (56%) | 201 (72%) |  | 55 (59%) | 377 (71%) |  |
| Female | 1 (25%) | 33 (43%) |  | 14 (44%) | 80 (28%) |  | 38 (41%) | 152 (29%) |  |
| **Age (years)** |  |  | **<0.001** |  |  | **<0.001** |  |  | **<0.001** |
| Median (IQR) | 44 (41, 47) | 70 (64, 74) |  | 46 (41, 48) | 66 (59, 72) |  | 43 (39, 46) | 66 (60, 73) |  |
| Range | 38, 49 | 54, 86 |  | 23, 49 | 50, 86 |  | 20, 49 | 50, 94 |  |
| **Smokers** |  |  | 0.824 |  |  | **0.018** |  |  | **0.002** |
| Never | 1 (25%) | 29 (43%) |  | 9 (29%) | 106 (39%) |  | 34 (39%) | 213 (42%) |  |
| Former | 2 (50%) | 24 (35%) |  | 7 (23%) | 101 (37%) |  | 18 (20%) | 171 (34%) |  |
| Current | 1 (25%) | 15 (22%) |  | 15 (48%) | 68 (25%) |  | 36 (41%) | 122 (24%) |  |
| Missing | 0 | 8 |  | 1 | 6 |  | 5 | 23 |  |
| **Body mass index (BMI)** |  |  | 0.101 |  |  | 0.140 |  |  | **0.002** |
| Median (IQR) | 43.0 (43.0, 43.0) | 26.4 (24.7, 28.6) |  | 24.5 (21.3, 27.2) | 25.5 (23.1, 28.4) |  | 23.9 (20.1, 26.9) | 25.3 (22.4, 28.7) |  |
| Range | 43.0, 43.0 | 16.4, 40.1 |  | 17.3, 30.5 | 16.8, 46.1 |  | 16.3, 36.0 | 13.3, 42.8 |  |
| Underweight | 0 (0%) | 2 (5.6%) | 0.216 | 3 (13%) | 5 (2.2%) | 0.076 | 8 (11%) | 19 (4.4%) | **0.023** |
| Normal weight | 0 (0%) | 8 (22%) |  | 11 (46%) | 94 (41%) |  | 39 (51%) | 186 (43%) |  |
| Overweight | 0 (0%) | 21 (58%) |  | 8 (33%) | 101 (44%) |  | 24 (32%) | 158 (37%) |  |
| Obesity | 1 (100%) | 5 (14%) |  | 2 (8.3%) | 31 (13%) |  | 5 (6.6%) | 66 (15%) |  |
| Missing | 3 | 40 |  | 8 | 50 |  | 17 | 100 |  |
| **Primary site** |  |  | 0.105 |  |  | 0.169 |  |  | 0.061 |
| Esophageal | 1 (25%) | 6 (7.9%) |  | 1 (3.1%) | 33 (12%) |  | 10 (11%) | 102 (19%) |  |
| GEJ | 1 (25%) | 4 (5.3%) |  | 11 (34%) | 63 (22%) |  | 13 (14%) | 93 (18%) |  |
| Gastric | 2 (50%) | 66 (87%) |  | 20 (63%) | 185 (66%) |  | 70 (75%) | 334 (63%) |  |
| **Adenocarcinoma subtype** |  |  | 0.645 |  |  | **<0.001** |  |  | **<0.001** |
| Poorly cohesive | 1 (25%) | 20 (27%) |  | 15 (47%) | 63 (23%) |  | 45 (48%) | 119 (23%) |  |
| Tubular | 0 (0%) | 8 (11%) |  | 2 (6.3%) | 15 (5.4%) |  | 11 (12%) | 18 (3.5%) |  |
| Mucinous | 0 (0%) | 3 (4.0%) |  | 1 (3.1%) | 11 (4.0%) |  | 7 (7.5%) | 11 (2.1%) |  |
| Other | 1 (25%) | 5 (6.7%) |  | 4 (13%) | 6 (2.2%) |  | 7 (7.5%) | 5 (1.0%) |  |
| NS | 2 (50%) | 39 (52%) |  | 10 (31%) | 183 (66%) |  | 23 (25%) | 364 (70%) |  |
| Unknown | 0 | 1 |  | 0 | 3 |  | 0 | 12 |  |
| **Visceral involvement** |  |  |  |  |  |  | 33/75 (44%) |  |  |
| **LAP** |  |  |  |  |  |  | 15/75 (20%) |  |  |
| **Peritoneal metastases** |  |  |  |  |  |  | 45/75 (60%) |  |  |
| **Bone metastases** |  |  |  |  |  |  | 6/75 (8.0%) |  |  |
| **Other metastatic sites** |  |  |  |  |  |  | 16/75 (21%) |  |  |
| **HER2 positive^*^** | 0/0 | 1/10 (10%) |  | 4/16 (25%) | 11/113 (9.7%) | 0.093 | 12/60 (20%) | 40/261 (15%) | 0.376 |
| **dMMR** | 0/0 | 1/5 (20%) |  | 0/10 (0%) | 6/48 (13%) | 0.577 | 2/22 (9.1%) | 3/71 (4.2%) | 0.589 |
| **EBV positive** | 0/0 | 0/3 (0%) |  | 0/0 | 0/15 (0%) |  | 0/7 (0%) | 2/32 (6.3%) |  |
| **PD-L1 positive^+^** | 0/0 | 2/3 (67%) |  | 1/7 (14%) | 7/37 (19%) | >0.999 | 5/17 (29%) | 26/69 (38%) | 0.525 |
| **Treated in clinical trials** | 0 (0%) | 3 (3.9%) |  | 3 (9.4%) | 35 (12%) | 0.780 | 16 (17%) | 74 (14%) | 0.416 |
| **Death** | 1 (25%) | 31 (41%) | 0.646 | 20 (63%) | 191 (68%) | 0.532 | 89 (96%) | 511 (97%) | 0.556 |

* HER-2 positivity is defined as IHC 3+ or IHC 2+ with ISH confirmed amplification

+ PD-L1 positivity is defined as follows: for adenocarcinoma, CPS ≥5; for squamous histology, TPS≥1%

Bold values indicate statistical significance (p < 0.05).

Abbreviations: dMMR, mismatch repair deficient; EBV, Epstein-Barr virus; ECOG, Eeastern Cooperative Oncology Group; GAPPS, gastric adenocarcinoma and proximal polyposis of the stomach; GEJ, gastroesophageal junction; HER-2, human epidermal growth factor receptor 2; IQR, interquartile range; LAP, lymphadenopathy; NOS, not otherwise specified; NS, not specified, PD-L1, programmed-death ligand 1

**Supplementary Table 3.** Overall survival estimates in the adenocarcinoma subgroup stratified by disease stage and localization.

|  |  | **N** | **Number**  **of deaths** | **Median OS** | **1-year OS rate** | **3-year OS rate** | **5-year OS rate** | **p-value** |
| --- | --- | --- | --- | --- | --- | --- | --- | --- |
| **Localized** | |  |  |  |  |  |  |  |
|  | All | 80 | 32 | 153.7 (73.3, —) | 88% (81, 96) | 75% (65, 85) | 61% (51, 74) |  |
|  | EO | 4 | 1 | 153.7 (—, —) | 100% (100, 100) | 100% (100, 100) | 100% (100, 100) | 0.335 |
|  | LO | 76 | 31 | — (57.2, —) | 88% (80, 96) | 73% (64, 84) | 59% (49, 72) |  |
| **Locally advanced, radically resected** | | | |  |  |  |  |  |
|  | All | 313 | 211 | 36.9 (32.6, 48.1) | 85% (81, 89) | 51% (46, 57) | 38% (33, 44) |  |
|  | EO | 32 | 20 | 55.2 (24.2, —) | 91% (81, 100) | 59% (45, 79) | 44% (29, 67) | 0.430 |
|  | LO | 281 | 191 | 36.1 (32.2, 44.4) | 84% (80, 89) | 50% (45, 57) | 37% (32, 44) |  |
| Esophageal/GEJ | EO | 12 | 6 | 57.7 (55.2, —) | 92% (77, 100) | 83% (65, 100) | 46% (22, 98) | 0.104 |
|  | LO | 96 | 67 | 31.1 (22.2, 48.5) | 81% (74, 89) | 46% (37, 58) | 33% (24, 45) |  |
| Gastric | EO | 20 | 14 | 26.0 (23.0, —) | 90% (78, 100) | 45% (28, 73) | 40% (23, 68) | 0.694 |
|  | LO | 185 | 124 | 37.7 (32.9, 52.2) | 86% (81, 91) | 53% (46, 60) | 39% (33, 47) |  |
| **Inoperable/Metastatic** | |  |  |  |  |  |  |  |
|  | All | 622 | 600 | 9.5 (8.4, 10.3) | 41% (37, 45) | 6.3% (4.6, 8.6) | 2.6% (1.6, 4.3) |  |
|  | EO | 93 | 89 | 9.1 (7.1, 10.5) | 32% (24, 43) | 8.0% (3.9, 17) | 2.7% (0.7, 10) | 0.913 |
|  | LO | 529 | 511 | 9.6 (8.3, 10.7) | 42% (38, 46) | 6.0% (4.3, 8.5) | 2.6% (1.5, 4.5) |  |
| Esophageal/GEJ | EO | 23 | 23 | 9.1 (5.1, 10.7) | 13% (4.5, 37) | 0% (—, —) | 0% (—, —) | **0.008** |
|  | LO | 195 | 189 | 9.9 (8.5, 12.6) | 44% (38, 52) | 6.8% (4.0, 11) | 2.2% (0.8, 6.0) |  |
| Gastric | EO | 70 | 66 | 9.1 (7.1, 14.7) | 39% (29, 52) | 11% (5.2, 22) | 3.6% (0.9, 14) | 0.320 |
|  | LO | 334 | 322 | 9.4 (7.4, 10.8) | 41% (36, 46) | 5.5% (3.5, 8.7) | 2.8% (1.5, 5.5) |  |

Bold values indicate statistical significance (p < 0.05).

Abbreviations: GEJ, gastroesophageal junction; EO, early-onset; LO, late-onset; OS, overall survival

**Supplementary Table 4.** Multivariable analysis for overall survival in the adenocarcinoma subgroup.

|  | **All adenocarcinomas** | | | | **Inoperable/Metastatic** | | | |
| --- | --- | --- | --- | --- | --- | --- | --- | --- |
|  | **N** | **Number**  **of deaths** | **HR (95% CI)** | **p-value** | **N** | **Number**  **of deaths** | **HR (95% CI)** | **p-value** |
| **Group** |  |  |  | 0.344 |  |  |  | 0.900 |
| Early-onset | 125 | 106 | — |  | 90 | 86 | — |  |
| Late-onset | 859 | 709 | 1.11 (0.90, 1.37) |  | 522 | 504 | 1.01 (0.81, 1.28) |  |
| **ECOG performance status** |  |  |  | **<0.001** |  |  |  | **<0.001** |
| 0 | 313 | 233 | — |  | 143 | 137 | — |  |
| 1 | 503 | 425 | 1.16 (0.99, 1.37) |  | 328 | 316 | 1.14 (0.93, 1.39) |  |
| 2 | 114 | 106 | 1.78 (1.41, 2.26) |  | 96 | 92 | 1.77 (1.36, 2.31) |  |
| 3-4 | 54 | 51 | 5.23 (3.83, 7.14) |  | 45 | 45 | 7.93 (5.57, 11.3) |  |
| **Sex** |  |  |  | **0.030** |  |  |  |  |
| Male | 678 | 576 | — |  |  |  |  |  |
| Female | 306 | 239 | 0.84 (0.72, 0.98) |  |  |  |  |  |
| **Adenocarcinoma subtype** |  |  |  | **0.001** |  |  |  |  |
| Poorly cohesive | 261 | 225 | — |  |  |  |  |  |
| Other | 723 | 590 | 0.76 (0.64, 0.89) |  |  |  |  |  |
| **Stage disease** |  |  |  | **<0.001** |  |  |  |  |
| Localized/Locally advanced, radically resected | 384 | 237 | — |  |  |  |  |  |
| Inoperable/Metastatic | 600 | 578 | 4.77 (4.02, 5.65) |  |  |  |  |  |

Bold values indicate statistical significance (p < 0.05).

Abbreviations: CI, confidence interval; ECOG, Eastern Cooperative Oncology Group; HR, hazard ratio

**Supplementary Table 5.** Univariable analysis for overall survival in the esophageal or GEJ adenocarcinoma subgroup.

|  | **All adenocarcinomas** | | | | **Inoperable/Metastatic** | | | |
| --- | --- | --- | --- | --- | --- | --- | --- | --- |
|  | **N** | **Number of deaths** | **HR (95% CI)** | **p-value** | **N** | **Number of deaths** | **HR (95% CI)** | **p-value** |
| **Group** |  |  |  | 0.328 |  |  |  | **0.015** |
| Early-onset | 37 | 30 | — |  | 23 | 23 | — |  |
| Late-onset | 301 | 262 | 1.21 (0.82, 1.77) |  | 195 | 189 | 0.55 (0.35, 0.86) |  |
| **ECOG performance status** |  |  |  | **<0.001** |  |  |  | **<0.001** |
| 0 | 93 | 71 | — |  | 43 | 40 | — |  |
| 1 | 183 | 160 | 1.50 (1.13, 1.99) |  | 123 | 120 | 1.19 (0.83, 1.70) |  |
| 2 | 37 | 37 | 3.49 (2.34, 5.23) |  | 33 | 33 | 2.47 (1.55, 3.94) |  |
| 3-4 | 18 | 18 | 3.49 (2.07, 5.88) |  | 14 | 14 | 5.88 (3.15, 11.0) |  |
| **Sex** |  |  |  | 0.815 |  |  |  | 0.093 |
| Male | 289 | 252 | — |  | 191 | 186 | — |  |
| Female | 49 | 40 | 0.96 (0.69, 1.34) |  | 27 | 26 | 1.45 (0.96, 2.19) |  |
| **Age (years)** | 338 | 292 | 1.01 (1.00, 1.02) | 0.232 | 218 | 212 | 1.0 (0.98, 1.01) | 0.430 |
| **Smokers** |  |  |  | 0.440 |  |  |  | 0.900 |
| Never | 104 | 89 | — |  | 71 | 69 | — |  |
| Former | 122 | 102 | 0.96 (0.72, 1.28) |  | 76 | 74 | 1.07 (0.77, 1.49) |  |
| Current | 97 | 88 | 1.15 (0.86, 1.55) |  | 63 | 61 | 1.01 (0.71, 1.42) |  |
| **Body mass index** |  |  |  | 0.068 |  |  |  | 0.607 |
| Normal weight | 93 | 81 | — |  | 63 | 62 | — |  |
| Underweight | 7 | 7 | 1.69 (0.78, 3.67) |  | 6 | 6 | 1.21 (0.52, 2.82) |  |
| Overweight | 108 | 95 | 1.05 (0.78, 1.41) |  | 70 | 68 | 0.91 (0.64, 1.28) |  |
| Obesity | 52 | 38 | 0.69 (0.47, 1.02) |  | 32 | 30 | 0.77 (0.50, 1.19) |  |
| **Adenocarcinoma subtype** |  |  |  | 0.916 |  |  |  | 0.968 |
| Poorly cohesive | 37 | 30 | — |  | 21 | 20 | — |  |
| Other | 292 | 254 | 0.98 (0.67, 1.43) |  | 192 | 187 | 0.99 (0.62, 1.57) |  |
| **Stage disease** |  |  |  | **<0.001** |  |  |  |  |
| Localized/Locally advanced, radically resected | 120 | 80 | — |  |  |  |  |  |
| Inoperable/Metastatic | 218 | 212 | 3.94 (2.98, 5.21) |  |  |  |  |  |

Bold values indicate statistical significance (p < 0.05).

Abbreviations: CI, confidence interval; ECOG, Eastern Cooperative Oncology Group; GEJ, gastroesophageal junction; HR, hazard ratio

**Supplementary Table 6.** Multivariable analysis for overall survival in the esophageal or GEJ adenocarcinoma subgroup.

|  | **All adenocarcinomas** | | | | **Inoperable/Metastatic** | | | |
| --- | --- | --- | --- | --- | --- | --- | --- | --- |
|  | **N** | **Number of deaths** | **HR (95% CI)** | **p-value** | **N** | **Number of deaths** | **HR (95% CI)** | **p-value** |
| **Group** |  |  |  | 0.690 |  |  |  | 0.057 |
| Early-onset | 36 | 29 | — |  | 22 | 22 | — |  |
| Late-onset | 295 | 257 | 0.92 (0.62, 1.37) |  | 191 | 185 | 0.62 (0.39, 0.99) |  |
| **ECOG performance status** |  |  |  | **<0.001** |  |  |  | **<0.001** |
| 0 | 93 | 71 | — |  | 43 | 40 | — |  |
| 1 | 183 | 160 | 1.20 (0.90, 1.60) |  | 123 | 120 | 1.16 (0.81, 1.67) |  |
| 2 | 37 | 37 | 2.41 (1.60, 3.62) |  | 33 | 33 | 2.28 (1.42, 3.67) |  |
| 3-4 | 18 | 18 | 3.74 (2.21, 6.33) |  | 14 | 14 | 5.83 (3.12, 10.9) |  |
| **Stage disease** |  |  |  | **<0.001** |  |  |  |  |
| Localized/Locally advanced, radically resected | 118 | 79 | — |  |  |  |  |  |
| Inoperable/Metastatic | 213 | 207 | 3.84 (2.87, 5.15) |  |  |  |  |  |

Bold values indicate statistical significance (p < 0.05).

Abbreviations: CI, confidence interval; ECOG, Eastern Cooperative Oncology Group; HR, hazard ratio

**Supplementary Table 7.** Univariable analysis for overall survival in the gastric adenocarcinoma subgroup.

|  | **All adenocarcinomas** | | | | **Inoperable/Metastatic** | | | |
| --- | --- | --- | --- | --- | --- | --- | --- | --- |
|  | **N** | **Number of deaths** | **HR (95% CI)** | **p-value** | **N** | **Number of deaths** | **HR (95% CI)** | **p-value** |
| **Group** |  |  |  | **0.049** |  |  |  | 0.316 |
| Early-onset | 92 | 80 | — |  | 70 | 66 | — |  |
| Late-onset | 585 | 471 | 0.78 (0.62, 0.99) |  | 334 | 322 | 1.14 (0.88, 1.49) |  |
| **ECOG performance status** |  |  |  | **<0.001** |  |  |  | **<0.001** |
| 0 | 225 | 167 | — |  | 100 | 97 | — |  |
| 1 | 327 | 271 | 1.38 (1.14, 1.67) |  | 205 | 196 | 1.14 (0.89, 1.45) |  |
| 2 | 79 | 71 | 2.45 (1.85, 3.24) |  | 63 | 59 | 1.55 (1.12, 2.14) |  |
| 3-4 | 38 | 35 | 4.04 (2.80, 5.83) |  | 31 | 31 | 10.0 (6.47, 15.6) |  |
| **Sex** |  |  |  | 0.110 |  |  |  | **0.028** |
| Male | 408 | 342 | — |  | 241 | 234 | — |  |
| Female | 269 | 209 | 0.87 (0.73, 1.03) |  | 163 | 154 | 0.80 (0.65, 0.98) |  |
| **Age (years)** | 677 | 551 | 1.00 (0.99, 1.01) | 0.893 | 404 | 388 | 1.01 (1.00, 1.02) | 0.114 |
| **Smokers** |  |  |  | 0.611 |  |  |  | 0.776 |
| Never | 288 | 230 | — |  | 176 | 167 | — |  |
| Former | 201 | 167 | 1.11 (0.91, 1.35) |  | 113 | 110 | 1.06 (0.84, 1.35) |  |
| Current | 160 | 131 | 1.03 (0.83, 1.27) |  | 95 | 92 | 1.09 (0.84, 1.41) |  |
| **Body mass index** |  |  |  | **0.007** |  |  |  | 0.456 |
| Normal weight | 245 | 213 | — |  | 162 | 158 | — |  |
| Underweight | 30 | 25 | 0.94 (0.62, 1.42) |  | 21 | 21 | 1.03 (0.65, 1.62) |  |
| Overweight | 204 | 153 | 0.71 (0.57, 0.87) |  | 112 | 103 | 0.82 (0.64, 1.06) |  |
| Obesity | 58 | 50 | 0.99 (0.73, 1.35) |  | 39 | 38 | 0.91 (0.64, 1.30) |  |
| **Adenocarcinoma subtype** |  |  |  | **0.004** |  |  |  | 0.371 |
| Poorly cohesive | 226 | 197 | — |  | 143 | 140 | — |  |
| Other | 444 | 347 | 0.77 (0.65, 0.92) |  | 254 | 241 | 0.91 (0.74, 1.12) |  |
| **Stage disease** |  |  |  | **<0.001** |  |  |  |  |
| Localized/Locally advanced, radically resected | 273 | 163 | — |  |  |  |  |  |
| Inoperable/Metastatic | 404 | 388 | 4.98 (4.09, 6.08) |  |  |  |  |  |

Bold values indicate statistical significance (p < 0.05).

Abbreviations: CI, confidence interval; ECOG, Eastern Cooperative Oncology Group; GEJ, gastroesophageal junction; HR, hazard ratio

**Supplementary Table 8.** Multivariable analysis for overall survival in the gastric adenocarcinoma subgroup.

|  | **All adenocarcinomas** | | | | **Inoperable/Metastatic** | | | |
| --- | --- | --- | --- | --- | --- | --- | --- | --- |
|  | **N** | **Number of deaths** | **HR (95% CI)** | **p-value** | **N** | **Number of deaths** | **HR (95% CI)** | **p-value** |
| **Group** |  |  |  | 0.102 |  |  |  | 0.120 |
| Early-onset | 89 | 77 | — |  | 68 | 64 | — |  |
| Late-onset | 573 | 460 | 1.23 (0.95, 1.58) |  | 324 | 312 | 1.24 (0.94, 1.65) |  |
| **ECOG performance status** |  |  |  | **<0.001** |  |  |  | **<0.001** |
| 0 | 223 | 165 | — |  | 98 | 95 | — |  |
| 1 | 324 | 268 | 1.16 (0.95, 1.41) |  | 202 | 193 | 1.17 (0.91, 1.50) |  |
| 2 | 77 | 69 | 1.57 (1.17, 2.10) |  | 61 | 57 | 1.54 (1.10, 2.16) |  |
| 3-4 | 38 | 35 | 5.24 (3.59, 7.65) |  | 31 | 31 | 10.1 (6.47, 15.7) |  |
| **Sex** |  |  |  | **0.004** |  |  |  | **0.021** |
| Male | 402 | 336 | — |  | 236 | 229 | — |  |
| Female | 260 | 201 | 0.77 (0.64, 0.92) |  | 156 | 147 | 0.78 (0.63, 0.96) |  |
| **Adenocarcinoma subtype** |  |  |  | **<0.001** |  |  |  | **0.049** |
| Poorly cohesive | 224 | 195 | — |  | 141 | 138 | — |  |
| Other | 438 | 342 | 0.69 (0.57, 0.84) |  | 251 | 238 | 0.79 (0.63, 1.00) |  |
| **Stage disease** |  |  |  | **<0.001** |  |  |  |  |
| Localized/Locally advanced, radically resected | 270 | 161 | — |  |  |  |  |  |
| Inoperable/Metastatic | 392 | 376 | 5.32 (4.31, 6.56) |  |  |  |  |  |

Bold values indicate statistical significance (p < 0.05).

Abbreviations: CI, confidence interval; ECOG, Eastern Cooperative Oncology Group; HR, hazard ratio
